# Editorial: Strategies for mitigating zoonotic influenza outbreaks: a comprehensive preparedness approach

**DOI:** 10.3389/fpubh.2025.1685224

**Published:** 2025-09-03

**Authors:** Sneha Vishwanath, Hazel Stewart, Sankaran Sandhya

**Affiliations:** ^1^Department of Veterinary Medicine, University of Cambridge, Cambridge, United Kingdom; ^2^Department of Pathology, University of Cambridge, Cambridge, United Kingdom; ^3^Independent Researcher, Bengaluru, India

**Keywords:** zoonotic influenza, pandemic preparedness and response, one-health approach, epidemiological studies, mathematical modeling, public health education, surveillance

## Introduction

The spillover of zoonotic influenza viruses into human populations and non-reservoir hosts such as cows, seals, and other animals continues to pose a significant threat to global public health. The emergence of strains like H5N1, H5N6, and H7N9 has not only disrupted societies but also tested—and at times overwhelmed—public health systems worldwide. With a growing human population, encroachment into wildlife habitats, climate change, and intensified human-animal interactions, the frequency and impact of zoonotic spillovers is expected to increase. To counter this looming threat, it is essential that we move beyond traditional, reactive approaches and adopt a robust, forward-looking preparedness strategy.

A key pillar in combating zoonotic influenza outbreaks is the One Health approach, which recognizes the interdependence of human, animal, and environmental health. This approach must form the foundation of any preparedness plan.

Key strategies under the one-health approach to mitigate zoonotic influenza outbreaks.

### Surveillance and early detection

Effective surveillance is vital for early detection and timely containment of zoonotic influenza outbreaks. Advancements in genomic sequencing, digital health systems, and data-sharing platforms have enabled real-time monitoring of viral mutations and disease burden, as evidenced during the COVID-19 pandemic. Possas et al., advocate for a shift from reactive approaches to a globally coordinated surveillance system which utilizes our advancements in technology. This is particularly relevant given the potential emergence of high-lethality influenza pandemics without available vaccines. Such surveillance systems must be integrated across veterinary, agricultural, and public health sectors. Early warning signals from poultry, swine, or wild birds can offer critical lead time for human health systems to prepare and respond.

For instance, a case study in India advocates the importance of integrating surveillance within existing public health frameworks. Abdulkader et al., detailed a comprehensive model in the Indian state of Tamil Nadu that combines clinical and epidemiological data with molecular diagnostics for effective early detection and response.

Similarly, a retrospective study by Zhou et al., on H5N6 outbreaks in Sichuan, China (2014–2024) highlights the importance of environmental surveillance in live poultry markets, farms, and migratory bird habitats, noting that human cases although rare were often fatal due to delayed treatment and co-morbidities.

### Risk assessment and modeling of transmission

Each zoonotic spillover—regardless of its initial scale—warrants rigorous risk assessment and modeling to forecast its trajectory within human populations and ecosystems. Islam et al., examined H5N1 transmission patterns in Bangladesh's domestic duck farming systems, highlighting the need for tailored surveillance and control strategies. Integrating local and national data sources, as shown by Li et al., ensures accurate assessment of non-seasonal influenza activity.

Modeling tools have also emerged as essential components of early warning systems. Perramon-Malavez et al., introduced a simplified tool adapted from the Moving Epidemic Method (MEM), predicting epidemic thresholds 6–7 days in advance for Influenza using the Effective Potential Growth (EPG) index. Jato-Espino et al., presented a spatial indicator system integrating ecological, environmental, and socio-economic data to identify high-risk transmission zones and support targeted interventions.

### Controlling transmission

Once human infections begin, transmission can be curtailed through two major approaches: (1) Isolation and quarantine, and (2) Vaccination campaigns. Isolation and quarantine, especially in the early stages of outbreaks, remain effective in the absence of vaccines. Kim et al., used machine learning to simulate the spread of MERS-CoV and found that targeted quarantining of cases and contacts outperformed mass isolation strategies in both efficiency and effectiveness. A strategy that could be extrapolated to zoonotic influenza outbreak. Once vaccines become available, population-wide immunization is the most efficient strategy. Xie et al., recommend optimizing vaccination site placements by minimizing queue times—a crucial lesson from the COVID-19 experience. Long wait times can deter participation and erode public trust.

In addition, computational advances have enabled the development of broadly protective vaccine antigens, as discussed by Possas et al., offering hope for variant-proof vaccine designs for Influenza.

### Public awareness and education

Public awareness is central to both prevention and control. Understanding zoonotic transmission pathways and the importance of vaccines is critical to public cooperation. Jia et al., highlight that low influenza vaccine coverage in children is often due to structural and informational gaps. They advocate for better parental education, easier access to vaccines at local clinics, and public awareness campaigns. Zhang et al., demonstrated that, even with low vaccination rates among children in Shanghai's Minhang District, vaccination efforts still prevented 6–17% of influenza cases and had substantial indirect benefits. This stresses the importance of community engagement and education in achieving high coverage and reducing disease burden.

To confront the growing threat of zoonotic influenza outbreaks, we must embrace a paradigm shift from reactive to proactive preparedness. By integrating the One Health approach across surveillance, modeling, transmission control, and public education, we can build resilient systems capable of early detection, rapid response, and sustainable prevention. Global health security hinges on our ability to anticipate and adapt. Coordinated action, investment in research, surveillance infrastructure and inclusive public health strategies will be vital. Our preparedness today will determine our resilience tomorrow.

